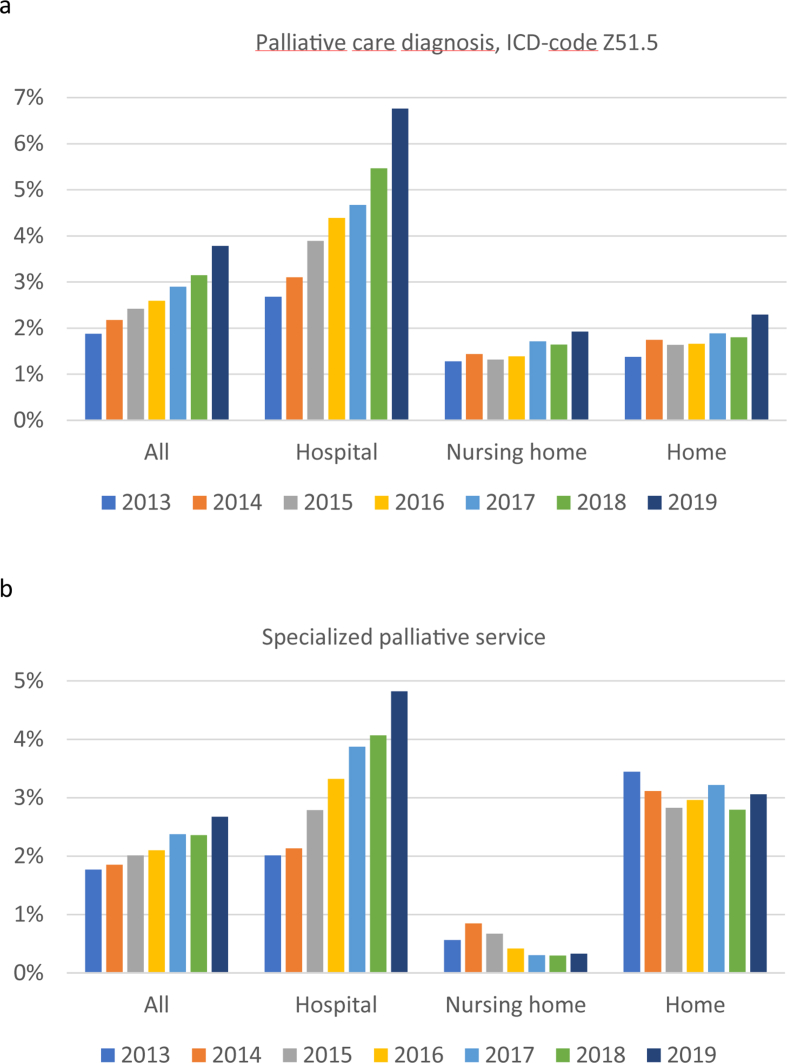# Registry study of cardiovascular death in Sweden 2013–2019: Home as place of death and specialized palliative care are the preserve of a minority

**DOI:** 10.1016/j.ijcrp.2024.200328

**Published:** 2024-09-02

**Authors:** Stina Nyblom, Joakim Öhlén, Cecilia Larsdotter, Anneli Ozanne, Carl Johan Fürst, Ragnhild Hedman

**Affiliations:** a Palliative Centre, Sahlgrenska University Hospital, Gothenburg, Sweden; b Institute of Medicine, Sahlgrenska Academy, University of Gothenburg, Gothenburg, Sweden; c Institute of Health and Care Sciences, Sahlgrenska Academy, University of Gothenburg, Gothenburg, Sweden; d Centre for Person-Centred Care (GPCC), Sahlgrenska Academy, University of Gothenburg, Gothenburg, Sweden; e Department of Nursing Science, Sophiahemmet University, Stockholm, Sweden; f Department of Neurology, Sahlgrenska University Hospital, Gothenburg, Sweden; g Faculty of Medicine, Department of Clinical Sciences, Lund, Sweden; h The Institute for Palliative Care, Respiratory Medicine, Allergology, and Palliative Medicine, Lund University, Lund, Sweden

**Keywords:** Cardiovascular diseases, Palliative care, Palliative medicine, Public health, Health services accessibility, Death, End-of-life

## Abstract

**Background:**

Palliative care needs in patients with cardiovascular diseases (CVD) are expected to increase. For the planning of equitable palliative care, it is important to understand where people with CVD die. The aim was to examine trends in place of death, associated factors including utilization of specialized palliative services, and to what extent longitudinal development is influenced by national policy.

**Methods:**

A population-level registry study of place of death for adults deceased due to CVD (n = 209 671) in Sweden 2013–2019. Linear regression analysis was applied.

**Results:**

The predominant place of death was nursing home (39.1 %) and hospital (37.6 %), followed by home (22.0 %). From 2013 to 2019 home deaths increased by 2.8 % and hospital deaths decreased by 3.0 %. An overall downward trend was found for dying in hospital compared to dying at home. With variations, this trend was seen in all healthcare regions and for all CVD types, except Stockholm and cerebrovascular disease, with no significant trend. Overall, but with cross-regional variations, 2.1 % utilized specialized palliative services, while 94.2 % had potential palliative care needs. Other variables significantly influencing the trend were age and having had an unplanned healthcare visit.

**Conclusion:**

Despite a slight positive trend, only a minority of people with CVD die in their own home. Regional variations in place of death and the low and varied utilization of specialized palliative services indicate inequity in access to palliative care. Hence, the impact of current national policies is questionable and calls for strengthening through inclusion of early palliative care in specific CVD policies.

## Abbreviations

CVD: cardiovascular disease
SPS: specialized palliative service

## 1 Introduction

Cardiovascular disease (CVD) are the leading cause of death worldwide, including Sweden, where CVD account for a third of all deaths [1]. Many people with CVD have palliative care needs, which is expected to increase due to an aging population and medical progress [[2], [3], [4], [5]]. For the planning and commissioning of equitable palliative care, it is important to understand where people with CVD spend the last part of their life and die, and to what extent the longitudinal development is influenced by national policy.

Palliative care is an approach that aims to improve the well-being of patients, as well as their loved ones, by preventing and alleviating problems in all aspects of life: physical, psychosocial and existential [6] and it can be delivered in specialized and non-specialized settings. Although palliative care arose out of, and still is mainly associated with, end-of-life care [7,8], it has now evolved into care with scientific support for the benefits of its application early in the course of the disease, as it can improve both quality and quantity of life [7,9,10].

Place of death, and the congruence between the patient's preferred and actual place of death, are seen globally as key outcome measures of the quality of palliative care in a country and can serve as a basis for decision-making in healthcare planning [11,12]. The preferred place of death is considered in Sweden, as in most international studies, to be the person's own home, but often, the place where people die does not align with their preferences, which can affect their end-of-life experience [[13], [14], [15], [16], [17], [18], [19]]. Although global estimates of place of death indicate that home predominates [20], most people in the Western world die in hospitals and nursing homes [16].

With the exception of specific CVD types [[21], [22], [23]], there are limited studies reporting the place of deaths and its associated factors for the whole population dying due to CVD. With an overall tradition of home death, in China, 77 % of CVD deaths occur at home [24]. Population-level studies from the US show that the primary site of CVD deaths has changed from hospital to 30.9 % dying at home [25]. Data from the first population-based place of death study in Sweden in 2012, showed that for CVD, 19.8 % died at home, 38.7 % in hospital and 40.2 % in nursing homes [26].

With heart failure being a common end pathway of many CVD [27], the need for palliative care in this disease group is great and will continue to increase [2]. However, palliative care remains underutilized in patients with CVD despite a high symptom burden in advanced disease, on par with, for example, metastatic cancer, and despite supportive evidence of its beneficial effect from multiple studies [[3], [4], [5],^28^]. This is often attributed to difficulties identifying palliative care needs, given the fluctuating and less clear prognostic trajectory of many CVD [4]. Early integration of palliative care for people with progressive CVD is therefore recognized as important for timely symptom relief, enhanced wellbeing and family member support. Accordingly, non-specialized palliative care is now recommended by the American Heart Association for all patients with heart failure [29,30], acknowledging the often sufficient support of non-specialized palliative care, which can and should be given by all healthcare professionals [31,32]. In Sweden, early palliative care is recommended for people with heart disease in the national guideline, initiated by professionals in palliative care [32]. The corresponding recommendation is not found in guidelines from the National Board of Health and Welfare [33] and, overall, early integrated palliative care is rarely practiced.

There is reason to assume that implementing policies that support earlier integration and expansion of palliative care not only improves patient well-being but may also reduce hospital deaths and increase the likelihood of dying at home [34,35]. In Sweden, the first national palliative care policies began to be implemented in 2013 [36,37]. As studies have shown that people with CVD often die in hospital, receive worse symptom relief and fewer end of life conversations than, for example, people who die of cancer, it is important to follow the eventual impact of these first national palliative care policies, in comparison to the first population-based place of death study in Sweden in 2012 [26]. This study was performed with the aim to identify longitudinal trends and variations in place of death for people with CVD as the underlying cause of death in Sweden between 2013 and 2019, at population level, and examine potential associations between place of death and individual characteristics, sociodemographics, healthcare service and utilization.

## 2 Methods

### 2.1 Study design

A nationwide, total population, registry study was conducted to explore trends in place of death among all adults (aged ≥18 years) with a CVD diagnosis as the underlying cause of death and a registered place of death in death certificates in Sweden 2013–2019. Data were obtained from the National Board of Health and Welfare (NBHW), Statistics Sweden, and the Swedish Register for Palliative Care (SRPC). Individual social security numbers were used to link data from different registers and were subsequently anonymized before being accessed by the researchers. The reason for choosing the included years was to cover a period from the implementation of the first national palliative care policy in Sweden (2013) and avoid possible influence on place of death from the COVID-19 pandemic (start 2020).

### 2.2 Setting

Over two million people live with CVD in Sweden and mortality increases with age. Healthcare in Sweden is tax-funded and regulated by law. The responsibility for healthcare is decentralized and divided, with the regions being responsible for hospitals and primary care and the municipalities for home care and nursing homes. Providers of healthcare, including care for older people, are either public or private, and the same regulations apply to both. Palliative care is integrated into the healthcare system and provided at various levels of care, including in hospitals, nursing homes and home care services, with non-specialized or specialized palliative services (SPS). Decentralized responsibility, together with cancer traditionally being the primary diagnosis for palliative care, often leads to variations in availability, as well as inequality between diagnoses when it comes to palliative care.

### 2.3 Study variables

Place of death was the primary dependent variable, with four alternatives: hospital (unspecified specialization), own home, nursing home (i.e. people registered as living in residential care settings and other forms of group dwellings), and other places (e.g. public places, roads, workplaces). Using ICD-10 codes, the underlying causes of CVD deaths (I00-I99) were divided into seven categories: Hypertensive diseases; Ischaemic heart diseases; Pulmonary heart disease and diseases of pulmonary circulation (henceforth called Pulmonary); Other forms of heart disease; Cerebrovascular diseases; Diseases of arteries, arterioles and capillaries; and Other (Table 1, Supplemental Tab. 1). As an indicator of official palliative care status, the ICD-10 code for palliative care (Z-51.5) was included. The Murtagh et al. model [38] was used for estimating palliative care needs within the population. The model is based on population-level death registration data, encompassing both underlying and contributory causes of death, and involves a more precise categorization of conditions relevant to palliative care, i.e. aligning with international policy [39]. Other included variables reported to affect place of death were socio-demographics and health service characteristics (healthcare region, urban/rural area) [40]. Healthcare utilization was reflected by frequency of hospital transfers and emergency department visits during the last month of life, and whether SPS were utilized at death (Table 1, Supplemental Tab. 1).

### 2.4 Analyses

Distribution of place of death and co-variables was displayed with descriptive statistics. Using linear regression, longitudinal trends in place of death and associated factors were investigated. With this model, a good fit could be shown to explain variations in place of death at population level, although the results were not continuous. Robust standard errors (HC3) were used to account for violations of the assumption of normality. Two separate analyses were performed: a) place of death in hospital as dependent variable for individuals residing in their own home (and dying in either hospitals or at home) and b) place of death in hospital as dependent variable for individuals older than 60 years residing in nursing homes (and dying in either hospitals or nursing homes). ‘Other places’ was excluded due to small numbers. Results are presented as percentage points change per year of people dying in hospital with 95 % confidence intervals (CIs). The coefficient of determination (R2) was used to summarize the strength of association at population (aggregate) level. This was calculated using linear regression on observed relative frequencies vs time. Interaction analyses were performed to evaluate the influence of covariates on longitudinal trends in place of death. All significance tests were two-sided and conducted at α = 0.001 significance level. Statistical analyses were performed using SAS/STAT Software, version 9.4 of the SAS System for Windows (SAS Institute Inc., Cary, NC).

## 3 Results

A total of 209 671 adults with CVD as the underlying cause of death were registered in Sweden in 2013–2019. The most common underlying causes of death were Ischaemic heart disease, Other forms of heart diseases, and Cerebrovascular diseases. At the time of death, the majority resided in an urban area, were ≥80 years of age, lived in single-person household and just over half were women. Distribution of place of death and other variables for the total CVD population are listed in Table 1.

**Table 1:** Distribution of place of CVD deaths by sex, age, CVD types and other variables in the total adult CVD population from 2013 to 2019.

Variables	% of total CVD deaths	Total CVD deaths (n = 209 671)	Home 22 %^a^ (n = 46 037)	Hospital 37.6 % (n = 78 738)	Nursing home 39.1 % (n = 82 004)	Other/unknown 1.4 % (n = 2892)
**Deaths per year**						
**2013**	15.0 %	31 406 (100.0 %)	6474 (20.6 %)	12 274 (39.1 %)	12 280 (39.1 %)	378 (1.2 %)
**2014**	14.6 %	30 694 (100.0 %)	6295 (20.5 %)	11 957 (39.0 %)	12 067 (39.3 %)	375 (1.2 %)
**2015**	14.5 %	30 340 (100.0 %)	6619 (21.8 %)	11 560 (38.1 %)	11 771 (38.8 %)	390 (1.3 %)
**2016**	14.5 %	30 416 (100.0 %)	6825 (22.4 %)	11 342 (37.3 %)	11 833 (38.9 %)	416 (1.4 %)
**2017**	14.3 %	30 048 (100.0 %)	6687 (22.3 %)	11 045 (36.8 %)	11 886 (39.6 %)	430 (1.4 %)
**2018**	14.0 %	29 307 (100.0 %)	6725 (22.9 %)	10 650 (36.3 %)	11 457 (39.1 %)	475 (1.6 %)
**2019**	13.1 %	27 460 (100.0 %)	6412 (23.4 %)	9910 (36.1 %)	10 710 (39.0 %)	428 (1.6 %)
**Sex**						
**Male**	48.5 %	101 747 (100.0 %)	26 960 (26.5 %)	41 819 (41.1 %)	30 715 (30.2 %)	2253 (2.2 %)
**Female**	51.5 %	107 924 (100.0 %)	19 077 (17.7 %)	36 919 (34.2 %)	51 289 (47.5 %)	639 (0.6 %)
**Age at death**						
**Age18**–**29**	0.1 %	189 (100.0 %)	85 (45.0 %)	90 (47.6 %)	6 (3.2 %)	8 (4.2 %)
**Age30**–**39**	0.2 %	374 (100.0 %)	158 (42.2 %)	184 (49.2 %)	8 (2.1 %)	24 (6.4 %)
**Age40**–**49**	0.8 %	1594 (100.0 %)	708 (44.4 %)	713 (44.7 %)	39 (2.4 %)	134 (8.4 %)
**Age50**–**59**	2.6 %	5388 (100.0 %)	2494 (46.3 %)	2295 (42.6 %)	208 (3.9 %)	391 (7.3 %)
**Age60**–**69**	7.8 %	16 456 (100.0 %)	7374 (44.8 %)	7069 (43.0 %)	1289 (7.8 %)	724 (4.4 %)
**Age70**–**79**	17.9 %	37 609 (100.0 %)	11 771 (31.3 %)	17 719 (47.1 %)	7323 (19.5 %)	796 (2.1 %)
**Age80**–**89**	38.2 %	79 980 (100.0 %)	14 378 (18.0 %)	32 294 (40.4 %)	32 715 (40.9 %)	593 (0.7 %)
**Age90+**	32.5 %	68 081 (100.0 %)	9069 (13.3 %)	18 374 (27.0 %)	40 416 (59.4 %)	222 (0.3 %)
**Underlying cause of death; CVD type (ICD-10)**						
**Hypertensive diseases (I10-I15)**	7.9 %	16 615 (100.0 %)	4271 (25.7 %)	3023 (18.2 %)	9212 (55.4 %)	109 (0.7 %)
**Ischaemic heart diseases (I20-I25)**	37.0 %	77 571 (100.0 %)	24 399 (31.5 %)	26 833 (34.6 %)	24 504 (31.6 %)	1835 (2.4 %)
**Pulmonary heart disease and diseases of pulmonary circulation (I26-I28) “Pulmonary”**	1.8 %	3702 (100.0 %)	814 (22.0 %)	2146 (58.0 %)	674 (18.2 %)	68 (1.8 %)
**Other forms of heart disease (I30-I52)**	27.7 %	58 108 (100.0 %)	10 021 (17.2 %)	21 247 (36.6 %)	26 315 (45.3 %)	525 (0.9 %)
**Cerebrovascular diseases (I60-I69)**	19.4 %	40 705 (100.0 %)	3538 (8.7 %)	19 148 (47.0 %)	17 868 (43.9 %)	151 (0.4 %)
**Diseases of arteries, arterioles and capillaries (I70-I79)**	5.4 %	11 297 (100.0 %)	2597 (23.0 %)	5466 (48.4 %)	3048 (27.0 %)	186 (1.6 %)
**Other (I95-I99, I00-I02, I05-I09, I80-I89)**	0.8 %	1673 (100.0 %)	397 (23.7 %)	875 (52.3 %)	383 (22.9 %)	18 (1.1 %)
**Marital status**^b^						
**Married**	28.1 %	58 815 (100.0 %)	12 527 (21.3 %)	29 584 (50.3 %)	15 610 (26.5 %)	1094 (1.9 %)
**Unmarried**	12.4 %	26 031 (100.0 %)	9209 (35.4 %)	8513 (32.7 %)	7565 (29.1 %)	744 (2.9 %)
**Widow**	44.0 %	92 319 (100.0 %)	14 650 (15.9 %)	28 720 (31.1 %)	48 504 (52.5 %)	445 (0.5 %)
**Divorced**	15.5 %	32 425 (100.0 %)	9616 (29.7 %)	11 889 (36.7 %)	10 317 (31.8 %)	603 (1.9 %)
**Educational attainment**^b^						
**No formal or elementary education**	44.5 %	93 255 (100.0 %)	17 000 (18.2 %)	33 074 (35.5 %)	42 422 (45.5 %)	759 (0.8 %)
**Lower secondary education**	7.7 %	16 131 (100.0 %)	4379 (27.1 %)	6107 (37.9 %)	5288 (32.8 %)	357 (2.2 %)
**Higher secondary education**	34.3 %	71 841 (100.0 %)	18 019 (25.1 %)	28 043 (39.0 %)	24 461 (34.0 %)	1318 (1.8 %)
**Higher education**	11.4 %	23 832 (100.0 %)	5678 (23.8 %)	9758 (40.9 %)	7976 (33.5 %)	420 (1.8 %)
**Birth country**						
**Sweden**	88.8 %	186 241 (100.0 %)	40 098 (21.5 %)	68 946 (37.0 %)	74 659 (40.1 %)	2538 (1.4 %)
**Outside Sweden**	11.2 %	23 430 (100.0 %)	5939 (25.3 %)	9792 (41.8 %)	7345 (31.3 %)	354 (1.5 %)
**Living situation**^b^						
**Home**	72.4 %	151 835 (100.0 %)	42 403 (27.9 %)	67 485 (44.4 %)	39 285 (25.9 %)	2662 (1.8 %)
**Nursing home**	20.5 %	42 894 (100.0 %)	1462 (3.4 %)	7245 (16.9 %)	34 115 (79.5 %)	72 (0.2 %)
**Other**	2.2 %	4515 (100.0 %)	1017 (22.5 %)	1635 (36.2 %)	1817 (40.2 %)	46 (1.0 %)
**Residing in urban area**^b^						
**NO**	11.1 %	23 235 (100.0 %)	6794 (29.2 %)	9414 (40.5 %)	6432 (27.7 %)	595 (2.6 %)
**Yes**	88.9 %	186 355 (100.0 %)	39 207 (21.0 %)	69 292 (37.2 %)	75 565 (40.5 %)	2291 (1.2 %)
**Health Care Region**^b^						
**Northern region**	10.7 %	22 474 (100.0 %)	4529 (20.2 %)	8318 (37.0 %)	9241 (41.1 %)	386 (1.7 %)
**Uppsala-Örebro region**	23.4 %	49 000 (100.0 %)	10 509 (21.4 %)	18 438 (37.6 %)	19 328 (39.4 %)	725 (1.5 %)
**Stockholm region**	16.6 %	34 821 (100.0 %)	7785 (22.4 %)	13 663 (39.2 %)	12 782 (36.7 %)	591 (1.7 %)
**Western region**	18.5 %	38 807 (100.0 %)	8532 (22.0 %)	14 650 (37.8 %)	15 091 (38.9 %)	534 (1.4 %)
**South-eastern region**	12.4 %	25 955 (100.0 %)	5645 (21.7 %)	8730 (33.6 %)	11 282 (43.5 %)	298 (1.1 %)
**Southern region**	18.4 %	38 534 (100.0 %)	9002 (23.4 %)	14 907 (38.7 %)	14 273 (37.0 %)	352 (0.9 %)
**Household situation**^b^						
**Single-person household**	59.1 %	12 3921 (100.0 %)	27 420 (22.1 %)	38 945 (31.4 %)	56 323 (45.5 %)	1233 (1.0 %)
**Multi-person household**	40.6 %	85 088 (100.0 %)	18 414 (21.6 %)	39 587 (46.5 %)	25 458 (29.9 %)	1629 (1.9 %)
**Children < 18 y**	2.0 %	4246 (100.0 %)	1026 (24.2 %)	1889 (44.5 %)	1166 (27.5 %)	165 (3.9 %)
**Potential palliative care needs**^c^						
**NO**	5.8 %	12 162 (100.0 %)	2862 (23.5 %)	5850 (48.1 %)	3253 (26.7 %)	197 (1.6 %)
**Yes**	94.2 %	197 509 (100.0 %)	43 175 (21.9 %)	72 888 (36.9 %)	78 751 (39.9 %)	2695 (1.4 %)
**No. of hospital transfers during the last month of life**						
**None**	56.5 %	118 385 (100.0 %)	39 504 (33.4 %)	14 398 (12.2 %)	62 007 (52.4 %)	2476 (2.1 %)
**One transfer**	29.2 %	61 317 (100.0 %)	4941 (8.1 %)	40 504 (66.1 %)	15 633 (25.5 %)	239 (0.4 %)
**Two or more transfers**	14.3 %	29 969 (100.0 %)	1592 (5.3 %)	23 836 (79.5 %)	4364 (14.6 %)	177 (0.6 %)
**No. of emergency department visits during the last month of life**						
**None**	69.9 %	146 500 (100.0 %)	40 685 (27.8 %)	35 875 (24.5 %)	67 363 (46.0 %)	2577 (1.8 %)
**One unplanned health care visit**	24.1 %	50 585 (100.0 %)	4363 (8.6 %)	33 824 (66.9 %)	12 154 (24.0 %)	244 (0.5 %)
**Two or more unplanned health care visits**	6.0 %	12 586 (100.0 %)	989 (7.9 %)	9039 (71.8 %)	2487 (19.8 %)	71 (0.6 %)
**Palliative care diagnosis; ICD-code Z51.5**						
**NO**	97.3 %	204 061 (100.0 %)	45 223 (22.2 %)	75 322 (36.9 %)	80 758 (39.6 %)	2758 (1.4 %)
**Yes**	2.7 %	5610 (100.0 %)	814 (14.5 %)	3416 (60.9 %)	1246 (22.2 %)	134 (2.4 %)
**Utilization of specialized palliative services at death**^d^						
**No**	97.9 %	205 160 (100.0 %)	44 630 (21.8 %)	76 198 (37.1 %)	81 600 (39.8 %)	2732 (1.3 %)
**Yes**	2.2 %	4511 (100.0 %)	1407 (31.2 %)	2540 (56.3 %)	404 (9.0 %)	160 (3.5 %)

a Percentages are row percentages.

b Missing data: Living situation 5 %; Educational attainment 2 %; Marital status <0.0 %; Urban area <0.0 %; Healthcare region <0.0 %; Household situation: <0.3 %. No missing data for remaining.

c Potential palliative care needs according to the Murtagh et al. model.^40^.

d Inpatient specialized palliative or hospice services, or specialized palliative home care services.

### 3.1 Distribution of place of death

Overall, the majority died in nursing homes or hospitals, and just over a fifth at home. From 2013 to 2019, the proportion of home deaths increased by 2.8 % and hospital deaths decreased by 3.0 %. The proportion of nursing home deaths was unchanged. All six healthcare regions showed an increase in home deaths, with the largest in the Southern region, which also had the largest decrease in hospital deaths. The smallest increase in home deaths was seen in Stockholm region. This was the only region not showing a decrease in hospital deaths. In 2019, Stockholm region showed the highest proportion of hospital deaths and the lowest for nursing home deaths with the reversed situation for the South-eastern region. The highest proportion of home deaths was found in the Southern region and the lowest in the Northern region (Fig. 1).

**Fig. 1:**
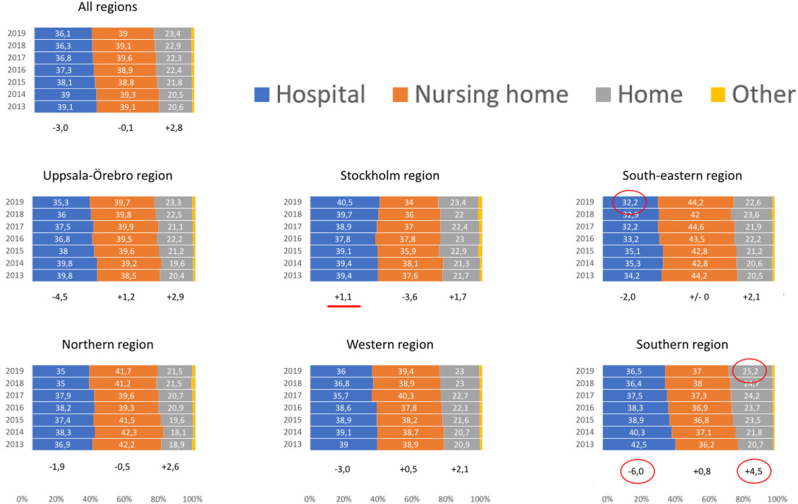
Overall and cross-regional distribution of CVD place of death in Sweden per year (2013–2019). Percentage change 2013–2019 is expressed below each regional column.

### 3.2 Trends in the place of death and influential factors

#### 3.2.1 Health services and sociodemographics

For the subgroup residing at home, that died either in hospital or at home, there was a small but significant downward trend in hospital deaths from 2013 to 2019 (−0.77, CI: 0.91,-0.62).

With variations, this downward trend was found in all healthcare regions, except Stockholm, and was most pronounced in the Southern region (−1.33, CI: 1.66,-1.00), which together with Uppsala-Örebro and the Northern region exceeded the overall trend. The trend was not consistent across age groups. Age 60–90+ showed a downward trend, age 40–59 showed no significant trend and age 18–39 showed an upward trend in hospital deaths. Other socio-demographic and health service variables showed no significant interaction with trend.

For those residing in a nursing home, aged ≥60 years and dying either in hospital or nursing home, there was a weak but significant overall downward trend in hospital deaths (−0.59, CI: 0.77,-0.40). Other sociodemographic and health service covariates showed no significant interaction with the trend.

For both home dwellers and nursing home residents, the highest proportion of hospital deaths for the respective covariate was 2019 found for born outside Sweden, having children <18, Stockholm region, residing in an urban area, with the addition of females, age 18–29, married, no formal education for home dwellers, and addition of men, age 60–69, divorced, higher education for nursing home residents (Fig. 2, Supplement Figs. 1 and 2).

**Fig. 2:**
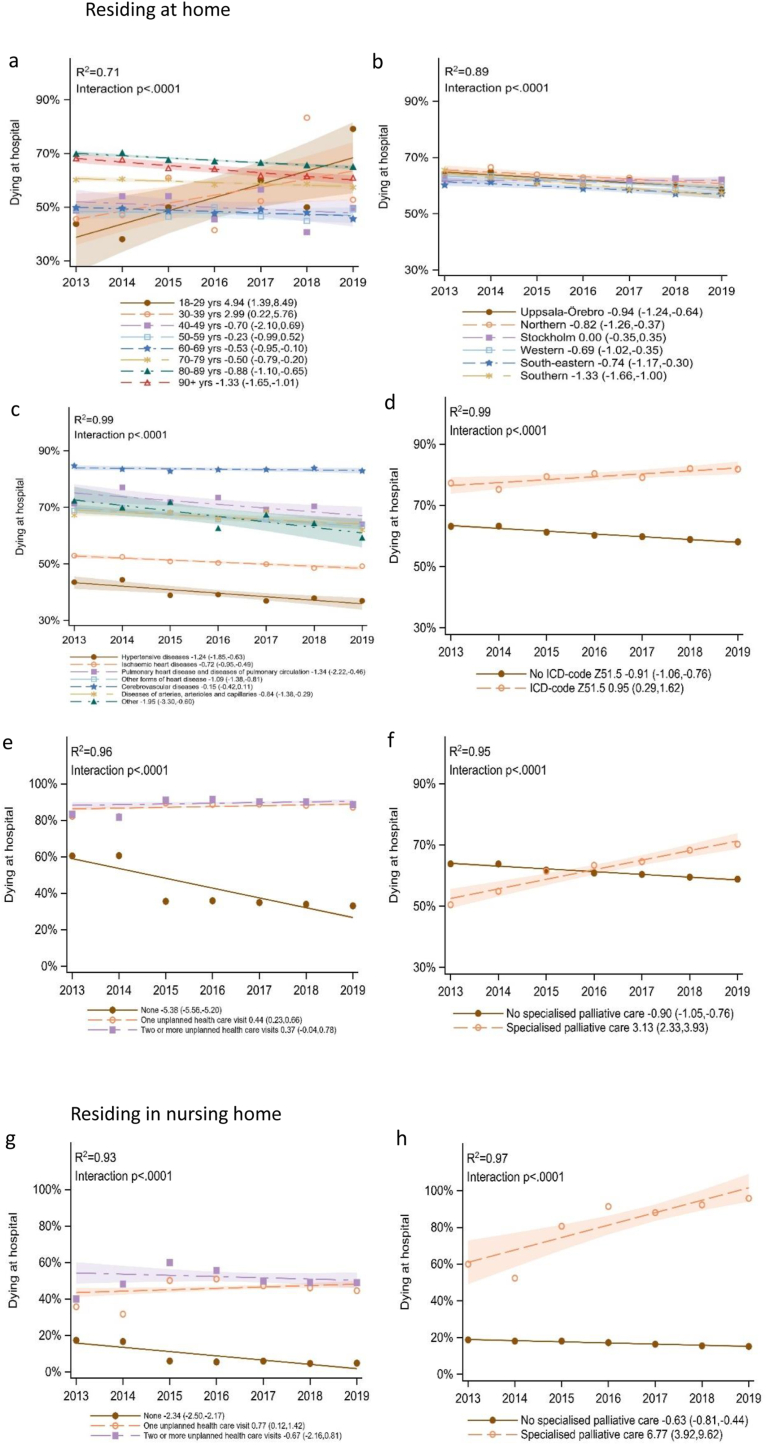
Longitudinal trends in place of CVD deaths based on linear regression. Residing at home and dying in either in hospital or at home (n = 109 888)): Age (Fig. 2a), Healthcare region (Fig. 2b), CVD type (Fig. 2c), Official palliative care status, ICD-code Z51.5 (Fig. 2d), Unplanned healthcare visit (Fig. 2e), Specialized palliative care (Fig. 2f). Residing in nursing home and dying in either in hospital or nursing home (n = 41196): Unplanned healthcare visit (Fig. 2g), Specialized palliative care (Fig. 2h).

#### 3.2.2 CVD type

Overall, Ischaemic heart diseases showed the highest proportion of home deaths and Cerebrovascular disease the lowest. Pulmonary showed the highest proportion of hospital deaths and Hypertensive disease the lowest. These latter two CVD types also represented the highest (Hypertensive) and lowest (Pulmonary) nursing home deaths (Table 1). The place of death for individual CVD types varied by health care region (Supplemental Table 2). Over the study period, an increase in the number of home deaths was seen in all individual CVD types, and a decrease in hospital deaths in all but Cerebrovascular disease. The largest change in both home and hospital deaths was found for Pulmonary (Supplemental Fig. 4).

For home dwellers, dying either in hospital or at home, a significant and variable downward trend in hospital deaths was found for all CVD types except Cerebrovascular diseases (no significant trend). Five CVD types exceeded the overall trend, most pronounced for Other. There were variations between CVD types in the proportion dying in hospital with the highest in Cerebrovascular and lowest in Hypertensive (Fig. 2).

For those residing in nursing home, CVD type showed no significant interaction with trend.

Pulmonary showed the highest proportions of hospital deaths and Hypertensive the lowest (Supplemental Fig. 2).

#### 3.2.3 Healthcare utilization

During the last month of life one or more hospital transfers and one or more unplanned emergency visits were common (Table 1). For both home dwellers and those living in nursing homes, emergency visits significantly interacted with trend resulting in an upward trend for hospital deaths. Hospital transfers did not significantly interact with the trend. Both hospital transfers and emergency visits showed a higher proportion of hospital deaths compared to the overall population, for both home dwellers and those living in nursing home (Fig. 2, Supplemental Fig. 3).

Overall, 2.1 % utilized specialist palliative services at death with a regional variation from 5.1 % (Stockholm) to 0.9 % (Western) (Table 2).

**Table 2:** Utilization of specialized palliative services at end-of-life: Overall distribution of place of CVD deaths by healthcare region.

	Home death		Hospital death		Nursing home death		Death in other place^a^	Specialized palliative care at death
Healthcare region	in pecialized palliative care service		in pecialized palliative care service		in pecialized palliative care service			
	No^a^	Yes^a^	No^a^	Yes^a^	No^a^	Yes^a^		
Northern region	4442 (19.8 %)	87 (0.4 %)	8215 (36.6 %)	103 (0.5 %)	9173 (40.8 %)	68 (0.3 %)	386 (1.7 %)	1.2 %
Uppsala-Örebro region	10361 (21.1 %)	148 (0.3 %)	17939 (36.6 %)	499 (1.0 %)	19271 (39.3 %)	57 (0.1 %)	725 (1.5 %)	1.4 %
Stockholm region	7128 (20.5 %)	657 (1.9 %)	12599 (36.2 %)	1064 (3.1 %)	12732 (36.6 %)	50 (0.1 %)	591 (1.7 %)	5.1 %
Western region	8426 (21.7 %)	106 (0.3 %)	14494 (37.3 %)	156 (0.4 %)	15003 (38.7 %)	88 (0.2 %)	534 (1.4 %)	0.9 %
South-eastern region	5400 (20.8 %)	245 (0.9 %)	8335 (32.1 %)	395 (1.5 %)	11209 (43.2 %)	73 (0.3 %)	298 (1.1 %)	2.7 %
Southern region	8838 (22.9 %)	164 (0.4 %)	14584 (37.8 %)	323 (0.8 %)	14205 (36.9 %)	68 (0.2 %)	352 (0.9 %)	1.4
**Total population**	**44630 (21.3 %)**	**1407 (0.7 %)**	**76198 (36.3 %)**	**2540 (1.2 %)**	**81600 (38.9 %)**	**404 (0.2 %)**	**2892 (1.4 %)**	**2.1 %**

Notes: ^a^ n and (row percentage).

In total, 2.7 % had the ICD-code Z51.5 for palliative care, while the majority were estimated to have potential palliative care needs (Table 1). For the total CVD population between 2013 and 2019, there was an increase in hospital deaths having a palliative care diagnosis as well as hospital deaths utilizing SPS (Supplemental Fig. 5). The individual CVD type that overall utilized the highest proportion of SPS was Other followed by Pulmonary, and the lowest proportion was seen for Hypertensive and Ischaemic heart diseases. Within the individual CVD types, the proportion utilizing SPS varied by region, with the greatest difference seen for Cerebrovascular disease (Supplemental Table 2).

An upward trend in hospital deaths was found for those utilizing SPS, significant for both home dwellers and those residing in a nursing home, as well as an upward trend for those having the ICD code for palliative care, only significant for home dwellers (Fig. 2, Supplemental Fig. 3).

## 4 Discussion

The present study of place of death for the CVD population in Sweden 2013–2019, showed a small but statistically significant trend towards a decrease in hospital deaths and an increase in deaths at home, as well as an indication of an increase in utilization of SPS at death in hospital, although still at a very low level. This development is in line with studies from other high-income countries for CVD deaths, as well as for the total population [25,39,41]. However, in comparison with the first population-based place of death study in Sweden 2012 [26], no convincing change was found that could be attributed to the implementation of the first national guidelines for palliative care in Sweden 2013. Home as place of death is the reality for only a minority of people who die due to CVD, and nursing homes or hospitals remain the most common place of death.

One could argue that the six years that have passed since the policy was implemented is too short a time to effect change. On the other hand, palliative care at the end of life has belonged to the highest priority group within the entire health care system since the Swedish national priority proposition was adopted in 1997 [42]. The results thus indicate continued challenges to promote equal access to adequate palliative care throughout the country.

The finding that very few patients had the ICD code Z51.5 for palliative care, in a CVD-population mostly >80 years of age, may reflect both that the code is underutilized and that few patients with CVD actually receive palliative care, non-specialized as well as specialized. Very few patients utilized SPS, similar to a previous study [43]. As we do not know the extent of non-specialized palliative care provided in the CVD population studied, we cannot determine whether this reflects provision of adequate non-specialized palliative care or underutilization and/or low access to SPS. However, the result indicates the latter, as international studies suggest that about 25 % of all annual deaths, regardless of diagnosis, need specialized palliative care [38,43]. The need might be even higher for CVD patients, especially older adults and those with severe disease for whom nearly 80 % prevalence of palliative care needs has been estimated [44].

The high proportion of hospital deaths in the CVD population can be partly explained by the nature of the disease. Many CVD deaths are due to heart attack and stroke, acute conditions that often require prompt medical intervention in hospital, where some also die. Consequences of stroke are often weakening and can hinder care and death at home which may be why cerebrovascular diseases showed the lowest proportion of home deaths, in accordance with previous studies [45]. Dyspnea and other complex symptoms are common in the late stages of CVD [46] and can be challenging in nonspecialized palliative settings, perhaps exemplified by patients with Pulmonary heart disease and diseases of pulmonary circulation being the most likely to die in hospital and the least likely to die in a nursing home. Moreover, many people who die due to cardiovascular disease in Sweden are older and live alone, which has been shown to increase the preference for dying in an institution [47].

The above examples highlight the difficulties in substituting hospital care for home care, a development many countries, including Sweden, strive for [[48], [49], [50]]. On the other hand, many CVD are chronic and could allow for planning for end of life and the active choice of home as the place of death. A known facilitator for this, and a service with demonstrated benefits for the CVD population, is specialized palliative care [16,19]. Thus, the overall low proportion of the CVD population found to utilize SPS in this study, despite the majority having potential palliative needs, indicates a lack of SPS as a facilitator and may be a reason for the limited number of home deaths. Contrary to this assumption, the study showed that having the ICD code for palliative care as well as utilizing SPS at death both showed a trend towards increased hospital deaths. Whether this reflects increased admissions to specialized palliative wards in hospitals cannot be discerned in this study and needs to be investigated further.

As shown in previous studies, people with CVD do not receive the same quality of palliative care as patients with cancer, despite the two being the leading causes of death with similar burdens of symptoms [46,51]. Traditionally, palliative care has focused on cancer and, although insufficient, cancer policy documents increasingly address palliative care. The same development has not yet been seen for CVD.

Taken together with the current findings, this indicates an external as well as internal inequity in access to palliative care for the CVD population; external in comparison with other disease groups with similar palliative care needs and internal due to regional variations in utilized SPS at death within the CVD population. The first national guidelines for palliative care do not seem to have had a major impact on the situation for people who die of CVD. If it is true that policy drives health care, now is the time to include palliative care in policies specific for CVD as well.

## 5 Limitations

Place of death is based on the four available options on the death certificate: hospital, home, nursing home and other. Whether the place of death is within a SPS is not specified. Utilization of SPS at death was based on data from the SRPC. This national quality register has a coverage rate of 56 % (2022) of all deaths in Sweden. However, it is known to cover close to all deaths in SPS and was thus used in coordination with death registers from the NBHW for calculations of the proportion of the CVD population that utilized SPS. The remaining individuals were assumed not to have utilized SPS, which may have resulted in an overestimation of deaths in this category. Moreover, there is no precise information available in Sweden about national or regional capacity for SPS, so the potential association of these factors with the place of death could therefore not be calculated. Several factors known to influence place of death were not included in this study such as preferred place of death, functional status, ethnicity, family carer support, home care use, socio-economics of the area, integration of home and hospital care services, and their extent of multidisciplinary teams [40].

## 6 Conclusion

For people with CVD as underlying cause of death in Sweden 2013–2019, there was a small overall increase in the proportion dying at home in all healthcare regions and for all CVD types, but still only just over a fifth die in their own home. An overall downward trend for hospital deaths was found in most regions, affected in the opposite direction by emergency department visits, utilization of SPS and, for home dwellers, younger age. Regional variations in place of death and low and varied utilization of SPS were found indicating inequity in access to palliative care and a need for stronger policy orientation towards earlier implementation of palliative care, non-specialized as well as specialized, to strengthen the care for the CVD population along the whole disease trajectory.

## Data availability

The data for this study are available from each register holder. Certain restrictions may apply. Programming codes are available from the authors upon reasonable request.

## Ethics approval

The Swedish Ethical Review Authority stated that the study could be conducted without ethical vetting as the sample consists of deceased individuals (no. 2019–05213, 2020-01758).

## Funding

This work was supported by the Swedish state under the agreement between the Swedish Government and the county councils, the ALF-agreement (ALFGBG-965941), The 10.13039/501100002794Swedish Cancer Society (grant no. 211580Pj01H) and The Local Research and Development Council Gothenburg and Södra Bohuslän, Sweden.

## CRediT authorship contribution statement

**Stina Nyblom:** Writing – original draft. **Joakim Öhlén:** Conceptualization. **Cecilia Larsdotter:** Conceptualization. **Anneli Ozanne:** Formal analysis, Data curation. **Carl Johan Fürst:** Conceptualization. **Ragnhild Hedman:** Formal analysis, Data curation.

## Declaration of competing interest

The authors report no relationships that could be construed as a conflict of interest.

## Figures and Tables

**figs1:**
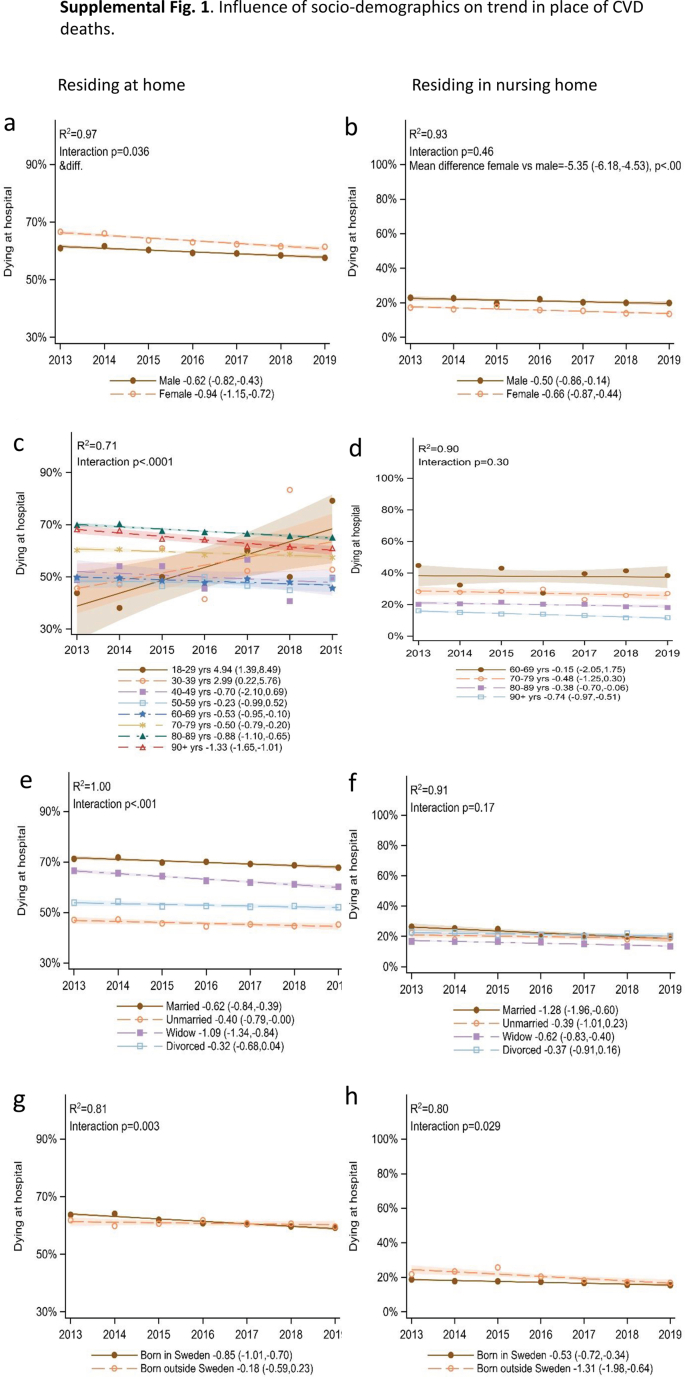


**figs2:**
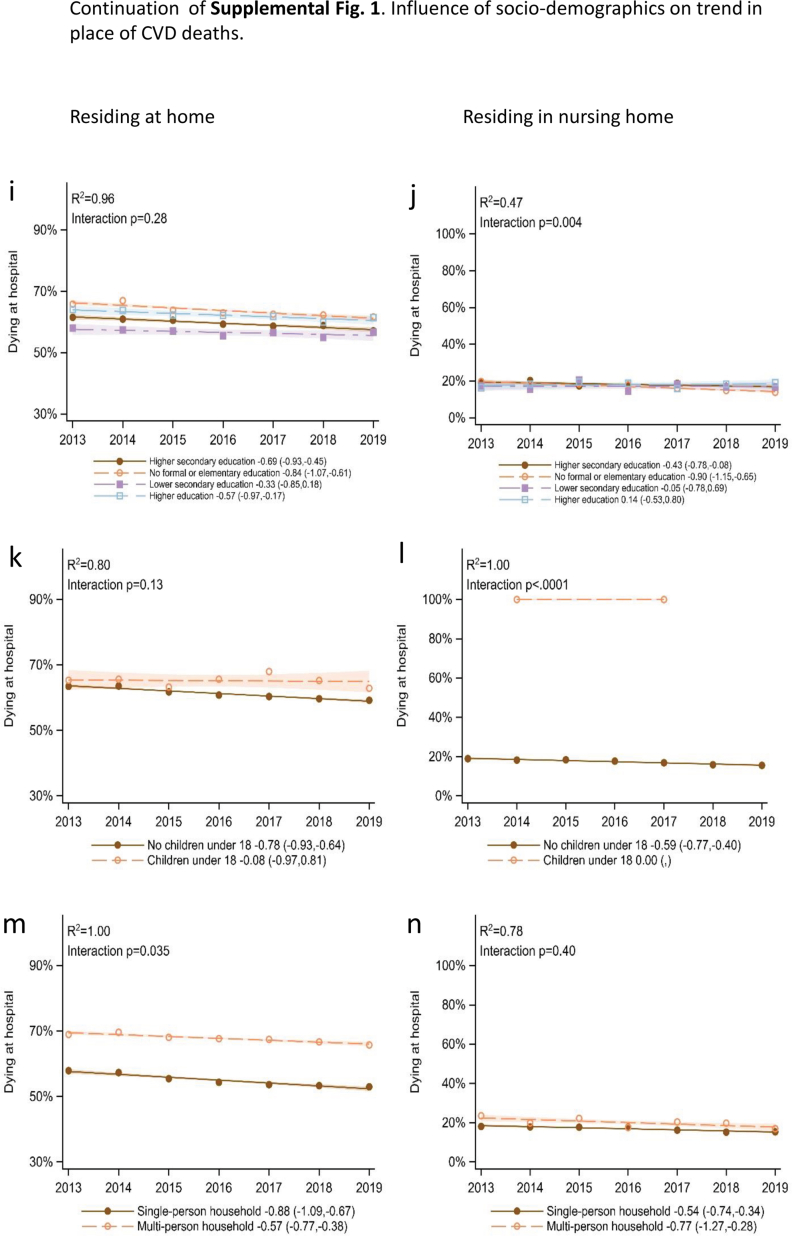


**figs3:**
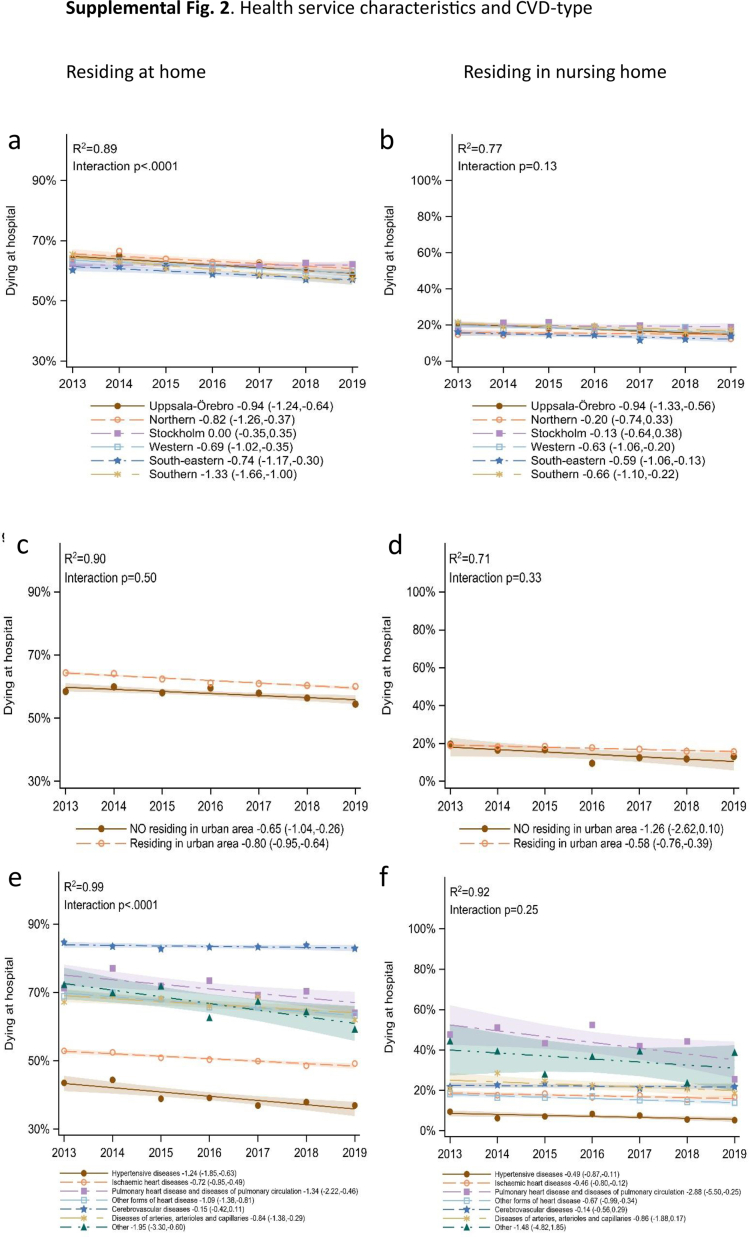


**figs4:**
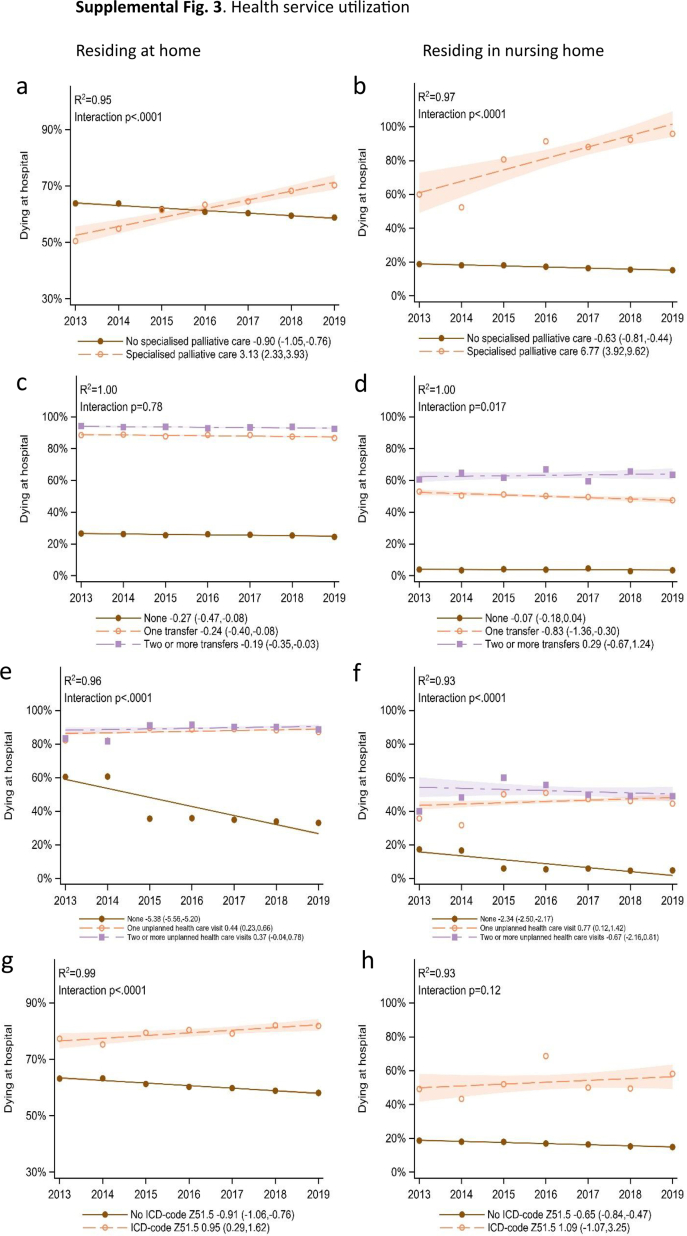


**figs5:**
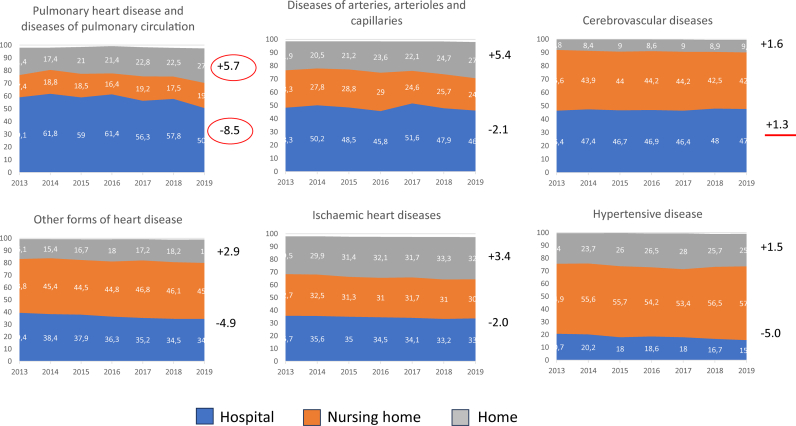


**figs6:**