# Supplementary material for: Registry study of cardiovascular death in Sweden 2013–2019: Home as place of death and specialized palliative care are the preserve of a minority
Source: Int J Cardiol Cardiovasc Risk Prev. 2024 Sep 2;23:200328. doi: 10.1016/j.ijcrp.2024.200328 (PMC11404052; doi:10.1016/j.ijcrp.2024.200328)
Supplement: Multimedia component 1 [file mmc1.docx]

**Supplemental Table 1**. Type of data, variables and categorisation as related to data sources

| **Type of data** | **Variables and categorisation** | **Data sources (register holders)** |
| --- | --- | --- |
| Outcome | Place of death  Home  Hospital  Nursing home  Other | Death certificate register (NBHW) ^2^ |
| Medical diagnoses | Types of CVD; based on ICD-10 codes ^1^  Hypertensive diseases (I10-I15)  Ischaemic heart diseases (I20-I25)  Pulmonary heart disease and diseases of pulmonary circulation (I26-I28)  Other forms of heart disease (I30-I52)  Cerebrovascular diseases (I60-I69)  Diseases of arteries, arterioles and capillaries (I70-I79)  Other (all other ICD-codes):  Acute rheumatic fever (I00-I02),  Chronic rheumatic heart diseases (I05-I09),  Diseases of veins, lymphatic vessels and lymph nodes,  not elsewhere classified (I80-I89),  Other and unspecified disorders o the circulatory system (I95-I99) | Death certificate register (NBHW) |
| Official palliative care status | Palliative care diagnosis  ICD-code Z51.5: Yes / No | Death certificate register (NBHW) |
| Socio-demographics | Sex  Male / Female  Age  Divided into 10-year categories  Marital status  Unmarried  Married  Widow  Divorced  Educational attainment  No formal or elementary education  Lower secondary education  Higher secondary education  Higher education  Birth country  Sweden / Other countries  Household situation  Living in single-person household: Yes / No  Children < 18 years in the household: Yes / No  Living situation  Home; own residence  Home; rented residence  Nursing home  Other  Residing in urban area: Yes / No | Death certificate register (NBHW)  National socioeconomic registers (StatS) ^3^ |
| Health service characteristics | National healthcare regions  Northern region  Uppsala-Örebro region  Stockholm region  West region  Southeast region  South region | Regional organisation of healthcare ^4^ |
| Healthcare service utilisation at the end-of-life | Hospital transfers during last month of life  None / One transfer / Two or more transfers  Emergency care during last month of life  None / One visit / Two or more visits  Specialised palliative care service at death ^5^  Specialised palliative care: Yes / No | Patient data register (NBHW)  Swedish Register for Palliative Care |

*Notes*. ^1^ Individual ICD-10 codes listed below. ^2^ National Board of Health and Welfare. ^3^ Statistics Sweden. ^4^ Well established nation-wide. ^5^ As related to place of death, the types of specialist palliative care services were: specialised palliative home care, specialised palliative hospital care, and municipality hospice care.
