# Supplementary material for: Registry study of cardiovascular death in Sweden 2013–2019: Home as place of death and specialized palliative care are the preserve of a minority
Source: Int J Cardiol Cardiovasc Risk Prev. 2024 Sep 2;23:200328. doi: 10.1016/j.ijcrp.2024.200328 (PMC11404052; doi:10.1016/j.ijcrp.2024.200328)
Supplement: Multimedia component 2 [file mmc2.docx]

**Supplemental Table 2**. Distribution of place of deaths from 2013 to 2019 by CVD type and healthcare region as related to receiving specialized palliative care at death or not. For each CVD type, the regions with the lowest proportion of deaths are highlighted in blue and the highest in yellow.

|  | | **Home death** | | **Hospital death** | | **Nursing home death** | | **Death in other place ^b^** |
| --- | --- | --- | --- | --- | --- | --- | --- | --- |
|  | | **Specialised palliative care services at home ^a^** | | **Specialised palliative care services at hospital ^a^** | | **Specialised palliative care services at nursing home ^a^** | |  |
| **CVD type** | **Healthcare region ^a^** | **No** | **Yes** | **No** | **Yes** | **No** | **Yes** |  |
| Hypertensive diseases | Northern | 379 (23.5%) | 5 (0.3%) | 276 (17.1%) | 3 (0.2%) | 933 (57.8%) | 1 (0.1%) | 16 (1.0%) |
|  | Uppsala-Örebro | 922 (22.3%) | 12 (0.3%) | 803 (19.5%) | 17 (0.4%) | 2326 (56.4%) | 3 (0.1%) | 44 (1.1%) |
|  | Stockholm | 959 (32.6%) | 26 (0.9%) | 499 (17.0%) | 46 (1.6%) | 1396 (47.5%) | 1 (0.0%) | 15 (0.5%) |
|  | Western | 866 (25.1%) | 11 (0.3%) | 549 (15.9%) | 4 (0.1%) | 1992 (57.8%) | 6 (0.2%) | 16 (0.5%) |
|  | South-eastern | 383 (21.4%) | 21 (1.2%) | 315 (17.6%) | 19 (1.1%) | 1041 (58.2%) | 3 (0.2%) | 6 (0.3%) |
|  | Southern | 680 (25.2%) | 6 (0.2%) | 489 (18.1%) | 3 (0.1%) | 1508 (55.9%) | 1 (0.0%) | 12 (0.4%) |
|  | **Total** | **4190 (25.2%)** | **81 (0.5%)** | **2931 (17.6%)** | **92 (0.6%)** | **9197 (55.4%)** | **15 (0.1%)** | **109 (0.7%)** |
| Ischaemic heart diseases | Northern | 2491 (28.6%) | 29 (0.3%) | 2976 (34.2%) | 32 (0.4%) | 2909 (33.4%) | 14 (0.2%) | 253 (2.9%) |
|  | Uppsala-Örebro | 5387 (29.9%) | 47 (0.3%) | 6087 (33.8%) | 93 (0.5%) | 5916 (32.9%) | 14 (0.1%) | 461 (2.6%) |
|  | Stockholm | 3848 (34.0%) | 152 (1.3%) | 4051 (35.8%) | 201 (1.8%) | 2721 (24.0%) | 8 (0.1%) | 333 (2.9%) |
|  | Western | 4608 (31.2%) | 39 (0.3%) | 4959 (33.6%) | 42 (0.3%) | 4731 (32.0%) | 26 (0.2%) | 362 (2.5%) |
|  | South-eastern | 2889 (28.0%) | 93 (0.9%) | 3091 (30.0%) | 115 (1.1%) | 3890 (37.7%) | 28 (0.3%) | 211 (2.0%) |
|  | Southern | 4751 (32.9%) | 42 (0.3%) | 5113 (35.5%) | 61 (0.4%) | 4235 (29.4%) | 9 (0.1%) | 210 (1.5%) |
|  | **Total** | **23997 (30.9%)** | **402 (0.5%)** | **26289 (33.9%)** | **544 (0.7%)** | **24405 (31.5%)** | **99 (0.1%)** | **1835 (2.4%)** |
| Pulmonary heart disease and diseases of pulmonary circulation | Northern | 59 (15.9%) | 3 (0.8%) | 230 (61.8%) | 5 (1.3%) | 66 (17.7%) | 1 (0.3%) | 8 (2.2%) |
|  | Uppsala-Örebro | 192 (22.2%) | 5 (0.6%) | 475 (55.0%) | 19 (2.2%) | 157 (18.2%) | 2 (0.2%) | 13 (1.5%) |
|  | Stockholm | 107 (17.6%) | 18 (3.0%) | 331 (54.4%) | 31 (5.1%) | 103 (16.9%) | 2 (0.3%) | 17 (2.8%) |
|  | Western | 113 (17.3%) | 6 (0.9%) | 408 (62.5%) | 2 (0.3%) | 101 (15.5%) | 8 (1.2%) | 15 (2.3%) |
|  | South-eastern | 119 (25.0%) | 5 (1.1%) | 232 (48.7%) | 9 (1.9%) | 101 (21.2%) | 2 (0.4%) | 8 (1.7%) |
|  | Southern | 179 (24.6%) | 8 (1.1%) | 392 (53.9%) | 10 (1.4%) | 130 (17.9%) | 1 (0.1%) | 7 (1.0%) |
|  | **Total** | **769 (20.8%)** | **45 (1.2%)** | **2070 (55.9%)** | **76 (2.1%)** | **658 (17.8%)** | **16 (0.4%)** | **68 (1.8%)** |
| Other forms of heart disease | Northern | 851 (15.2%) | 41 (0.7%) | 1890 (33.8%) | 40 (0.7%) | 2665 (47.7%) | 38 (0.7%) | 63 (1.1%) |
|  | Uppsala-Örebro | 2328 (17.3%) | 62 (0.5%) | 4735 (35.1%) | 191 (1.4%) | 6034 (44.7%) | 24 (0.2%) | 115 (0.9%) |
|  | Stockholm | 1223 (11.1%) | 352 (3.2%) | 3678 (33.5%) | 463 (4.2%) | 5114 (46.5%) | 18 (0.2%) | 144 (1.3%) |
|  | Western | 1737 (16.9%) | 36 (0.3%) | 3858 (37.4%) | 65 (0.6%) | 4485 (43.5%) | 34 (0.3%) | 92 (0.9%) |
|  | South-eastern | 1251 (18.7%) | 90 (1.3%) | 2053 (30.7%) | 107 (1.6%) | 3116 (46.6%) | 31 (0.5%) | 35 (0.5%) |
|  | Southern | 1950 (17.7%) | 94 (0.9%) | 4038 (36.6%) | 123 (1.1%) | 4714 (42.7%) | 40 (0.4%) | 76 (0.7%) |
|  | **Total** | **9346 (16.1%)** | **675 (1.2%)** | **20258 (34.9%)** | **989 (1.7%)** | **26130 (45.0%)** | **185 (0.3%)** | **525 (0.9%)** |
| Cerebrovascular diseases | Northern | 350 (7.4%) | 5 (0.1%) | 2117 (44.6%) | 17 (0.4%) | 2232 (47.0%) | 7 (0.1%) | 17 (0.4%) |
|  | Uppsala-Örebro | 793 (8.5%) | 15 (0.2%) | 4395 (47.1%) | 139 (1.5%) | 3940 (42.2%) | 11 (0.1%) | 47 (0.5%) |
|  | Stockholm | 398 (6.0%) | 66 (1.0%) | 3099 (47.0%) | 251 (3.8%) | 2736 (41.5%) | 15 (0.2%) | 31 (0.5%) |
|  | Western | 661 (8.8%) | 6 (0.1%) | 3572 (47.7%) | 32 (0.4%) | 3194 (42.6%) | 6 (0.1%) | 22 (0.3%) |
|  | South-eastern | 456 (8.8%) | 21 (0.4%) | 1952 (37.9%) | 116 (2.2%) | 2589 (50.2%) | 7 (0.1%) | 15 (0.3%) |
|  | Southern | 756 (10.3%) | 7 (0.1%) | 3347 (45.5%) | 102 (1.4%) | 3119 (42.4%) | 11 (0.1%) | 19 (0.3%) |
|  | **Total** | **3418 (8.4%)** | **120 (0.3%)** | **18491 (45.4%)** | **657 (1.6%)** | **17811 (43.8%)** | **57 (0.1%)** | **151 (0.4%)** |
| Diseases of arteries, arterioles and capillaries | Northern | 280 (21.5%) | 2 (0.2%) | 653 (50.2%) | 5 (0.4%) | 330 (25.3%) | 7 (0.5%) | 25 (1.9%) |
|  | Uppsala-Örebro | 655 (23.3%) | 4 (0.1%) | 1259 (44.9%) | 34 (1.2%) | 812 (28.9%) | 2 (0.1%) | 41 (1.5%) |
|  | Stockholm | 528 (25.3%) | 36 (1.7%) | 807 (38.6%) | 58 (2.8%) | 609 (29.1%) | 6 (0.3%) | 47 (2.2%) |
|  | Western | 369 (20.3%) | 2 (0.1%) | 988 (54.2%) | 8 (0.4%) | 426 (23.4%) | 6 (0.3%) | 23 (1.3%) |
|  | South-eastern | 242 (18.8%) | 10 (0.8%) | 572 (44.4%) | 22 (1.7%) | 421 (32.7%) | 1 (0.1%) | 21 (1.6%) |
|  | Southern | 463 (23.4%) | 5 (0.3%) | 1037 (52.3%) | 20 (1.0%) | 423 (21.4%) | 5 (0.3%) | 28 (1.4%) |
|  | **Total** | **2538 (22.5%)** | **59 (0.5%)** | **5319 (47.1%)** | **147 (1.3%)** | **3021 (26.7%)** | **27 (0.2%)** | **186 (1.6%)** |
| Other | Northern | 32 (21.3%) | 2 (1.3%) | 73 (48.7%) | 1 (0.7%) | 38 (25.3%) | 0 (0.0%) | 4 (2.7%) |
|  | Uppsala-Örebro | 84 (22.8%) | 3 (0.8%) | 185 (50.1%) | 6 (1.6%) | 86 (23.3%) | 1 (0.3%) | 4 (1.1%) |
|  | Stockholm | 65 (23.5%) | 7 (2.5%) | 134 (48.4%) | 14 (5.1%) | 53 (19.1%) | 0 (0.0%) | 4 (1.4%) |
|  | Western | 72 (22.4%) | 6 (1.9%) | 160 (49.8%) | 3 (0.9%) | 74 (23.1%) | 2 (0.6%) | 4 (1.2%) |
|  | South-eastern | 60 (24.4%) | 5 (2.0%) | 120 (48.8%) | 7 (2.8%) | 51 (20.7%) | 1 (0.4%) | 2 (0.8%) |
|  | Southern | 59 (19.0%) | 2 (0.6%) | 168 (54.2%) | 4 (1.3%) | 76 (24.5%) | 1 (0.3%) | 0 (0.0%) |
|  | **Total** | **372 (22.2%)** | **25 (1.5%)** | **840 (50.2%)** | **35 (2.1%)** | **378 (22.6%)** | **5 (0.3%)** | **18 (1.1%)** |
| Total | Northern | 4442 (19.8%) | 87 (0.4%) | 8215 (36.6%) | 103 (0.5%) | 9173 (40.8%) | 68 (0.3%) | 386 (1.7%) |
|  | Uppsala-Örebro | 10361 (21.1%) | 148 (0.3%) | 17939 (36.6%) | 499 (1.0%) | 19271 (39.3%) | 57 (0.1%) | 725 (1.5%) |
|  | Stockholm | 7128 (20.5%) | 657 (1.9%) | 12599 (36.2%) | 1064 (3.1%) | 12732 (36.6%) | 50 (0.1%) | 591 (1.7%) |
|  | Western | 8426 (21.7%) | 106 (0.3%) | 14494 (37.3%) | 156 (0.4%) | 15003 (38.7%) | 88 (0.2%) | 534 (1.4%) |
|  | South-eastern | 5400 (20.8%) | 245 (0.9%) | 8335 (32.1%) | 395 (1.5%) | 11209 (43.2%) | 73 (0.3%) | 298 (1.1%) |
|  | Southern | 8838 (22.9%) | 164 (0.4%) | 14584 (37.8%) | 323 (0.8%) | 14205 (36.9%) | 68 (0.2%) | 352 (0.9%) |
|  | **Total** | **44630 (21.3%)** | **1407 (0.7%)** | **76198 (36.3%)** | **2540 (1.2%)** | **81600 (38.9%)** | **404 (0.2%)** | **2892 (1.4%)** |
| ^a^ n and (row percentages)  ^b^ workplace, road, etc. | | | | | | | | |
