# Supplementary material for: Registry study of cardiovascular death in Sweden 2013–2019: Home as place of death and specialized palliative care are the preserve of a minority
Source: Int J Cardiol Cardiovasc Risk Prev. 2024 Sep 2;23:200328. doi: 10.1016/j.ijcrp.2024.200328 (PMC11404052; doi:10.1016/j.ijcrp.2024.200328)
Supplement: Multimedia component 3 [file mmc3.docx]

**Supplemental Table 3**. Proportion of Home, Hospital, and Nursing home deaths per year and as related to the six healthcare regions.

Proportion of home deaths

| **Health Care Region** | 2013 | 2014 | 2015 | 2016 | 2017 | 2018 | 2019 | Diff 2013-2019 |
| --- | --- | --- | --- | --- | --- | --- | --- | --- |
| **Uppsala-Örebro region** | 1452 (20.4%) | 1370 (19.6%) | 1530 (21.2%) | 1591 (22.2%) | 1475 (21.1%) | 1560 (22.5%) | 1531 (23.3%) | +2,9 |
| **Northern region** | 652 (18.9%) | 597 (18.1%) | 622 (19.6%) | 695 (20.9%) | 658 (20.7%) | 662 (21.5%) | 643 (21.5%) | +2,6 |
| **Stockholm region** | 1128 (21.7%) | 1125 (21.3%) | 1160 (22.9%) | 1186 (23.0%) | 1110 (22.4%) | 1053 (22.0%) | 1023 (23.4%) | +1,7 |
| **Western region** | 1217 (20.9%) | 1164 (20.7%) | 1197 (21.6%) | 1231 (22.1%) | 1257 (22.7%) | 1270 (23.0%) | 1196 (23.0%) | +2,1 |
| **South-eastern region** | 821 (20.5%) | 787 (20.6%) | 796 (21.2%) | 827 (22.2%) | 827 (21.9%) | 848 (23.6%) | 739 (22.6%) | +2,1 |
| **Southern region** | 1200 (20.7%) | 1243 (21.8%) | 1310 (23.5%) | 1293 (23.7%) | 1349 (24.2%) | 1332 (24.7%) | 1275 (25.2%) | +4,5 |

Proportion of hospital deaths

| **Health Care Region** | 2013 | 2014 | 2015 | 2016 | 2017 | 2018 | 2019 | Diff 2013-2019 |
| --- | --- | --- | --- | --- | --- | --- | --- | --- |
| **Uppsala-Örebro region** | 2838 (39.8%) | 2774 (39.8%) | 2738 (38.0%) | 2643 (36.8%) | 2625 (37.5%) | 2500 (36.0%) | 2320 (35.3%) | -4,5 |
| **North region** | 1271 (36.9%) | 1262 (38.3%) | 1189 (37.4%) | 1268 (38.2%) | 1206 (37.9%) | 1076 (35.0%) | 1046 (35.0%) | -1,9 |
| **Stockholm region** | 2051 (39.4%) | 2078 (39.4%) | 1978 (39.1%) | 1953 (37.8%) | 1928 (38.9%) | 1902 (39.7%) | 1773 (40.5%) | +1,1 |
| **West region** | 2269 (39.0%) | 2196 (39.1%) | 2157 (38.9%) | 2144 (38.6%) | 1979 (35.7%) | 2033 (36.8%) | 1872 (36.0%) | -3,0 |
| **Southeast region** | 1372 (34.2%) | 1350 (35.3%) | 1321 (35.1%) | 1239 (33.2%) | 1217 (32.2%) | 1180 (32.9%) | 1051 (32.2%) | -2,0 |
| **South region** | 2466 (42.5%) | 2291 (40.3%) | 2169 (38.9%) | 2091 (38.3%) | 2084 (37.5%) | 1959 (36.4%) | 1847 (36.5%) | -6,0 |

Proportion of nursing home deaths

| **Health Care Region** | 2013 | 2014 | 2015 | 2016 | 2017 | 2018 | 2019 | Diff 2013-2019 |
| --- | --- | --- | --- | --- | --- | --- | --- | --- |
| **Uppsala-Örebro region** | 2740 (38.5%) | 2737 (39.2%) | 2855 (39.6%) | 2833 (39.5%) | 2794 (39.9%) | 2760 (39.8%) | 2609 (39.7%) | +1,2 |
| **North region** | 1452 (42.2%) | 1396 (42.3%) | 1317 (41.5%) | 1304 (39.3%) | 1259 (39.6%) | 1268 (41.2%) | 1245 (41.7%) | -0,5 |
| **Stockholm region** | 1956 (37.6%) | 2009 (38.1%) | 1819 (35.9%) | 1953 (37.8%) | 1834 (37.0%) | 1724 (36.0%) | 1487 (34.0%) | -3,6 |
| **West region** | 2262 (38.9%) | 2173 (38.7%) | 2119 (38.2%) | 2103 (37.8%) | 2236 (40.3%) | 2149 (38.9%) | 2049 (39.4%) | +0,5 |
| **Southeast region** | 1773 (44.2%) | 1639 (42.8%) | 1609 (42.8%) | 1622 (43.5%) | 1684 (44.6%) | 1510 (42.0%) | 1445 (44.2%) | +0,0 |
| **South region** | 2097 (36.2%) | 2113 (37.1%) | 2050 (36.8%) | 2017 (36.9%) | 2076 (37.3%) | 2046 (38.0%) | 1874 (37.0%) | +0,8 |
