# Supplementary material for: Registry study of cardiovascular death in Sweden 2013–2019: Home as place of death and specialized palliative care are the preserve of a minority
Source: Int J Cardiol Cardiovasc Risk Prev. 2024 Sep 2;23:200328. doi: 10.1016/j.ijcrp.2024.200328 (PMC11404052; doi:10.1016/j.ijcrp.2024.200328)
Supplement: Multimedia component 4 [file mmc4.docx]

**Supplemental Table 4**. Cross-regional population characteristics.

| **Variable** |  | | | | | | |
| --- | --- | --- | --- | --- | --- | --- | --- |
|  | **Total (n=209591)** | **Uppsala-Örebro region (reference) (n=49000)** | **Northern region (n=22474)** | **Stockholm region (n=34821)** | **Western region (n=38807)** | **South-eastern region (n=25955)** | **Southern region (n=38534)** |
| **Sex** |  |  |  |  |  |  |  |
| **Male** | 101679 (48.5%) | 23855 (48.7%) | 11291 (50.2%) | 16571 (47.6%) | 19026 (49.0%) | 12376 (47.7%) | 18560 (48.2%) |
| **Female** | 107912 (51.5%) | 25145 (51.3%) | 11183 (49.8%) | 18250 (52.4%) | 19781 (51.0%) | 13579 (52.3%) | 19974 (51.8%) |
| **Age at death** |  |  |  |  |  |  |  |
| **Age60-69 (reference)** | 16430 (7.8%) | 3924 (8.0%) | 1838 (8.2%) | 2996 (8.6%) | 2967 (7.6%) | 1758 (6.8%) | 2947 (7.6%) |
| **Age18-29** | 189 (0.1%) | 54 (0.1%) | 21 (0.1%) | 29 (0.1%) | 31 (0.1%) | 24 (0.1%) | 30 (0.1%) |
| **Age30-39** | 373 (0.2%) | 76 (0.2%) | 31 (0.1%) | 77 (0.2%) | 71 (0.2%) | 47 (0.2%) | 71 (0.2%) |
| **Age40-49** | 1588 (0.8%) | 366 (0.7%) | 155 (0.7%) | 295 (0.8%) | 322 (0.8%) | 157 (0.6%) | 293 (0.8%) |
| **Age50-59** | 5377 (2.6%) | 1231 (2.5%) | 587 (2.6%) | 1016 (2.9%) | 1037 (2.7%) | 583 (2.2%) | 923 (2.4%) |
| **Age70-79** | 37590 (17.9%) | 8917 (18.2%) | 4335 (19.3%) | 6467 (18.6%) | 6704 (17.3%) | 4403 (17.0%) | 6764 (17.6%) |
| **Age80-89** | 79969 (38.2%) | 18974 (38.7%) | 9323 (41.5%) | 12263 (35.2%) | 14771 (38.1%) | 10081 (38.8%) | 14557 (37.8%) |
| **Age90+** | 68075 (32.5%) | 15458 (31.5%) | 6184 (27.5%) | 11678 (33.5%) | 12904 (33.3%) | 8902 (34.3%) | 12949 (33.6%) |
| **Disease of the circulary system** |  |  |  |  |  |  |  |
| **Hypertensive diseases (I10-I15)** | 16613 (7.9%) | 4127 (8.4%) | 1613 (7.2%) | 2942 (8.4%) | 3444 (8.9%) | 1788 (6.9%) | 2699 (7.0%) |
| **Ischaemic heart diseases (I20-I25)** | 77528 (37.0%) | 18005 (36.7%) | 8704 (38.7%) | 11314 (32.5%) | 14767 (38.1%) | 10317 (39.7%) | 14421 (37.4%) |
| **Pulmonary heart disease and diseases of pulmonary circulation (I26-I28)** | 3700 (1.8%) | 863 (1.8%) | 372 (1.7%) | 609 (1.7%) | 653 (1.7%) | 476 (1.8%) | 727 (1.9%) |
| **Other forms of heart disease (I30-I52)** | 58094 (27.7%) | 13489 (27.5%) | 5588 (24.9%) | 10992 (31.6%) | 10307 (26.6%) | 6683 (25.7%) | 11035 (28.6%) |
| **Cerebrovascular diseases (I60-I69)** | 40691 (19.4%) | 9340 (19.1%) | 4745 (21.1%) | 6596 (18.9%) | 7493 (19.3%) | 5156 (19.9%) | 7361 (19.1%) |
| **Diseases of arteries, arterioles and capillaries (I70-I79)** | 11292 (5.4%) | 2807 (5.7%) | 1302 (5.8%) | 2091 (6.0%) | 1822 (4.7%) | 1289 (5.0%) | 1981 (5.1%) |
| **Other : I95-I99, I00-I02, I05-I09, I80-I89.** | 1673 (0.8%) | 369 (0.8%) | 150 (0.7%) | 277 (0.8%) | 321 (0.8%) | 246 (0.9%) | 310 (0.8%) |
| **Living conditions, Number of children under 18** |  |  |  |  |  |  |  |
| **No children under 18** | 204763 (98.0%) | 48031 (98.2%) | 22036 (98.2%) | 33629 (97.1%) | 37879 (97.8%) | 25505 (98.4%) | 37683 (98.2%) |
| **Children under 18** | 4246 (2.0%) | 879 (1.8%) | 406 (1.8%) | 999 (2.9%) | 838 (2.2%) | 415 (1.6%) | 709 (1.8%) |
| **Living in single-person household** |  |  |  |  |  |  |  |
| **Single-person household** | 123921 (59.3%) | 29148 (59.6%) | 12819 (57.1%) | 20520 (59.3%) | 22863 (59.1%) | 15598 (60.2%) | 22973 (59.8%) |
| **Multi-person household** | 85088 (40.7%) | 19762 (40.4%) | 9623 (42.9%) | 14108 (40.7%) | 15854 (40.9%) | 10322 (39.8%) | 15419 (40.2%) |
| **Potential palliative care needs** |  |  |  |  |  |  |  |
| **NO (reference)** | 12157 (5.8%) | 2983 (6.1%) | 1379 (6.1%) | 2246 (6.5%) | 1972 (5.1%) | 1412 (5.4%) | 2165 (5.6%) |
| **Potential palliative care needs** | 197434 (94.2%) | 46017 (93.9%) | 21095 (93.9%) | 32575 (93.5%) | 36835 (94.9%) | 24543 (94.6%) | 36369 (94.4%) |
| **Year of Death** |  |  |  |  |  |  |  |
| **2013** | 31394 (15.0%) | 7126 (14.5%) | 3442 (15.3%) | 5201 (14.9%) | 5818 (15.0%) | 4007 (15.4%) | 5800 (15.1%) |
| **2014** | 30678 (14.6%) | 6978 (14.2%) | 3298 (14.7%) | 5277 (15.2%) | 5611 (14.5%) | 3825 (14.7%) | 5689 (14.8%) |
| **2015** | 30326 (14.5%) | 7205 (14.7%) | 3176 (14.1%) | 5064 (14.5%) | 5552 (14.3%) | 3759 (14.5%) | 5570 (14.5%) |
| **2016** | 30409 (14.5%) | 7176 (14.6%) | 3319 (14.8%) | 5163 (14.8%) | 5558 (14.3%) | 3730 (14.4%) | 5463 (14.2%) |
| **2017** | 30024 (14.3%) | 7007 (14.3%) | 3178 (14.1%) | 4958 (14.2%) | 5543 (14.3%) | 3774 (14.5%) | 5564 (14.4%) |
| **2018** | 29307 (14.0%) | 6939 (14.2%) | 3076 (13.7%) | 4785 (13.7%) | 5531 (14.3%) | 3591 (13.8%) | 5385 (14.0%) |
| **2019** | 27453 (13.1%) | 6569 (13.4%) | 2985 (13.3%) | 4373 (12.6%) | 5194 (13.4%) | 3269 (12.6%) | 5063 (13.1%) |
| **Marital status** |  |  |  |  |  |  |  |
| **Married** | 58815 (28.1%) | 13628 (27.8%) | 6375 (28.4%) | 9189 (26.4%) | 11028 (28.4%) | 7458 (28.7%) | 11137 (28.9%) |
| **Unmarried** | 26031 (12.4%) | 6043 (12.3%) | 3229 (14.4%) | 4696 (13.5%) | 4761 (12.3%) | 2943 (11.3%) | 4359 (11.3%) |
| **Widow** | 92319 (44.0%) | 21783 (44.5%) | 9897 (44.0%) | 14162 (40.7%) | 16989 (43.8%) | 12239 (47.2%) | 17249 (44.8%) |
| **Divorced** | 32425 (15.5%) | 7546 (15.4%) | 2973 (13.2%) | 6774 (19.5%) | 6029 (15.5%) | 3315 (12.8%) | 5788 (15.0%) |
| **Educational attainment** |  |  |  |  |  |  |  |
| **Higher secondary education (reference)** | 71818 (35.0%) | 16180 (33.6%) | 7989 (35.9%) | 13901 (41.4%) | 12760 (33.6%) | 7898 (31.1%) | 13090 (34.8%) |
| **No formal or elementary education** | 93245 (45.5%) | 23642 (49.1%) | 10718 (48.1%) | 10045 (29.9%) | 18034 (47.5%) | 13337 (52.4%) | 17469 (46.4%) |
| **Lower secondary education** | 16125 (7.9%) | 3408 (7.1%) | 1400 (6.3%) | 3423 (10.2%) | 3096 (8.2%) | 1868 (7.3%) | 2930 (7.8%) |
| **Higher education** | 23812 (11.6%) | 4936 (10.2%) | 2166 (9.7%) | 6198 (18.5%) | 4044 (10.7%) | 2333 (9.2%) | 4135 (11.0%) |
| **Residing in urban area** |  |  |  |  |  |  |  |
| **NO (reference)** | 23235 (11.1%) | 6415 (13.1%) | 4162 (18.5%) | 1598 (4.6%) | 4439 (11.4%) | 3033 (11.7%) | 3588 (9.3%) |
| **Residing in urban area** | 186355 (88.9%) | 42585 (86.9%) | 18312 (81.5%) | 33223 (95.4%) | 34368 (88.6%) | 22921 (88.3%) | 34946 (90.7%) |
| **Country of birth** |  |  |  |  |  |  |  |
| **Born in Sweden (reference)** | 186198 (88.8%) | 44031 (89.9%) | 21158 (94.1%) | 28262 (81.2%) | 34341 (88.5%) | 23972 (92.4%) | 34434 (89.4%) |
| **Born outside Sweden** | 23393 (11.2%) | 4969 (10.1%) | 1316 (5.9%) | 6559 (18.8%) | 4466 (11.5%) | 1983 (7.6%) | 4100 (10.6%) |
| **Place of death within a specialised palliative care facility** |  |  |  |  |  |  |  |
| **No specialised palliative care** | 205080 (97.8%) | 48296 (98.6%) | 22205 (98.8%) | 32925 (94.6%) | 38449 (99.1%) | 25241 (97.2%) | 37964 (98.5%) |
| **Specialised palliative care** | 4511 (2.2%) | 704 (1.4%) | 269 (1.2%) | 1896 (5.4%) | 358 (0.9%) | 714 (2.8%) | 570 (1.5%) |
| **Palliative care diagnosis ICD-code Z51.5** |  |  |  |  |  |  |  |
| **No ICD-code Z51.5** | 203982 (97.3%) | 48431 (98.8%) | 22036 (98.1%) | 32793 (94.2%) | 38364 (98.9%) | 24639 (94.9%) | 37719 (97.9%) |
| **ICD-code Z51.5** | 5609 (2.7%) | 569 (1.2%) | 438 (1.9%) | 2028 (5.8%) | 443 (1.1%) | 1316 (5.1%) | 815 (2.1%) |
| For categorical variables n (%) is presented. For continuous variables Mean (SD) /Median (Min; Max) / n= is presented. | | | | | | | |
